# Hollow core photonic crystal fiber-assisted Raman spectroscopy as a tool for the detection of Alzheimer’s disease biomarkers

**DOI:** 10.1117/1.JBO.25.7.077001

**Published:** 2020-07-02

**Authors:** Pinkie J. Eravuchira, Martina Banchelli, Cristiano D’Andrea, Marella de Angelis, Paolo Matteini, Israel Gannot

**Affiliations:** a Tel Aviv University, Department of Biomedical Engineering, Faculty of Engineering, Tel Aviv, Israel; b Institute of Applied Physics “NelloCarrara,” National Research Council, Sesto Fiorentino, Italy; c Johns Hopkins University, Department of Electrical and Computer Engineering, Baltimore, Maryland, United States

**Keywords:** Alzheimer’s, amyloid β-peptide, fiber-enhanced Raman spectroscopy, liquid biopsy, surface-enhanced Raman spectroscopy

## Abstract

**Significance:** Alzheimer’s disease (AD) is an irreversible and progressive disorder that damages brain cells and impairs the cognitive abilities of the affected. Developing a sensitive and cost-effective method to detect Alzheimer’s biomarkers appears vital in both a diagnostic and therapeutic perspective.

**Aim:** Our goal is to develop a sensitive and reliable tool for detection of amyloid β (1-42) peptide (Aβ42), a major AD biomarker, using fiber-enhanced Raman spectroscopy (FERS).

**Approach:** A hollow core photonic crystal fiber (HCPCF) was integrated with a conventional Raman spectroscopic setup to perform FERS measurements. FERS was then coupled with surface-enhanced Raman spectroscopy (SERS) to further amplify the Raman signal thanks to a combined FERS-SERS assay.

**Results:** A minimum 20-fold enhancement of the Raman signal of Aβ42 as compared to a conventional Raman spectroscopy scheme was observed using the HCPCF-based light delivery system. The signal was further boosted by decorating the fiber core with gold bipyramids generating an additional SERS effect, resulting in an overall 200 times amplification.

**Conclusions:** The results demonstrate that the use of an HCPCF-based platform can provide sharp and intense Raman signals of Aβ42, in turn paving the way toward the development of a sensitive label-free detection tool for early diagnosis of AD.

## 1 Introduction

Alzheimer’s disease (AD) is a chronic, progressive, neurodegenerative disorder that affects several million of people worldwide.^1^^–^^4^ AD is considered as one of the leading causes of dementia, and it is ranked as the fifth major cause of death. AD is pathologically characterized by the deposition of extracellular plaques composed of amyloid β-peptide (Aβ) and the aggregation of tau protein as intracellular neurofibrillary tangles in the brain.^5^^–^^10^ Specifically, the entorhinalcortex and hippocampus, which are brain areas dealing with cognitive abilities, are critically affected by the deposition of plaques and tangles in such a way that cognitive disabilities, including memory impairment, difficulties with reasoning and solving problems, and challenges with time and space, are among the prime distinctive symptoms of AD. The current antemortem AD diagnosis is based on monitoring the mental decline and on neuropsychological and laboratory tests. Nevertheless, the conclusive diagnosis of AD is still challenging requiring an autopsy substantiation of disease pathology.

Molecular biomarkers are explicit indicators of a specific health and disease state, and their detection is crucial for diagnosis, monitoring of disease progression, and treatment. Protein species such as Aβ and tau protein are associated with AD and recognized as main biomarkers of AD. Studies evidenced that biochemical changes leading to progressive accumulation of such biomarkers begin even 15 to 20 years before the first symptom appears. Although as of now there is no definitive cure for AD, several medications that may slow down the progress of AD are already available in the market.^11^^,^^12^ Therefore, a presymptomatic diagnosis of AD is fundamental as it would aid to maintain normal brain activity. Tremendous efforts are being made to develop effective and ultrasensitive methods for the detection of AD biomarkers. Amyloid positron emission tomography (PET) represents a powerful and efficient technique for imaging of Aβ in the brain at an early stage of the disease progression.^13^^–^^16^ Nonetheless, high costs, limited accessibility, and health hazards restrain this technique as a gold standard for AD diagnosis. On the other hand, detection of trace amounts of Aβ and tau protein shed into the cerebrospinal fluid (CSF) or other accessible biofluids is now recognized as a potential alternative for early AD diagnosis.^17^^,^^18^ On this basis, developing a cost-effective, reliable, and sensitive technique that allows for sensitive detection of AD biomarkers at an early stage will be a huge advancement in the field of AD diagnosis and treatment. Numerous techniques such as liquid chromatography, enzyme-linked immunosorbent assay (ELISA), mass spectrometry, flow cytometry, electrochemical sensing, surface plasmon resonance, fluorescence, and Raman spectroscopy are being studied and tested for the detection of AD biomarkers.^6^^,^^19^^–^^27^ Among the optical methods, Raman spectroscopy and its related techniques are being demonstrated as a reliable tool providing label-free fingerprint information from biomolecules including AD biomarkers and misfolded protein species associated with neurodegenerative conditions.^28^^–^^32^ High chemical specificity, noninvasiveness, reproducibility, and rapidness make present Raman-based methods a promising option for the detection of AD.^33^^–^^35^ Nevertheless, the low efficiency of the Raman scattering process and high-fluorescence background limit this technique for the detection of biomolecules, especially when the concentration of the analyte is reduced. The weak Raman scattering can be compensated by either increasing the excitation laser power or by increasing the number of analyte molecules that interact with laser light. However, increasing the laser power is not an ideal solution, especially for heat-sensitive molecules, as are most of the biomolecules. Fiber-enhanced Raman spectroscopy (FERS) can address the above limitations by increasing the interaction between analyte molecules and the excitation laser. FERS is a method that integrates a microstructured optical fiber with a conventional Raman spectroscopic system and has gained remarkable attention as a detection technique for applications ranging from biosensing to health monitoring.^34^^–^^46^ Among the microstructured optical fibers, hollow core photonic crystal fiber (HCPCF) has proved to be a potential detection tool, especially for the analysis of trace amounts of analyte molecules. HCPCF is a novel microstructured photonic crystal fiber that has a central hollow core surrounded by a periodic arrangement of air holes. An incident laser of a definite wavelength gets confined into the fiber core due to the photonic band-gap effect of the periodic array cladding and the light spreads through the fiber core with very low attenuation. FERS based on HCPCF has proven convenient for a broad range of applications dealing with the detection of liquid and gaseous samples.^36^^–^^38^ In this case, the analyte is collected inside the fiber holes, i.e., making HCPCF act as a miniaturized sample container, whereas an increased interaction between laser light and analyte molecules is achieved within the HCPCF core. Compared to a conventional Raman spectroscopic approach, Raman scattering is highly enhanced due to the unique FERS geometry, which, in turn, improves the detection sensitivity. Additionally, a stable light coupling can be achieved using HCPCF, as the transmission spectrum of HCPCF remains unaffected even with the bending of the optical fiber.

Exploiting the optical properties of the HCPCF, we developed an ultrasensitive and label-free sensing system based on FERS for the detection of Aβ. For this purpose, an HCPCF-based light delivery system was properly assembled for the Raman measurements. In addition, to study the possibility to further increase the signal, the FERS system was integrated with surface-enhanced Raman spectroscopy (SERS), as extra expedient to circumvent the sensitivity limitations of Raman scattering. Low sampling volume, compact size, sensitivity, rapidness, and cost-effectiveness make the proposed label-free platform a promising detection tool for the early diagnosis of AD pathology.

## 2 Methods

### 2.1 Reagents and Chemicals

Myoglobin (Mb) from horse skeletal muscle and Amyloid β (1-42) peptide (Aβ42) were purchased from Sigma-Aldrich and used as received. Aβ42 solution was prepared by dissolving Aβ42 powder in 50 mM NaOH and finally diluting in phosphate-buffered saline (PBS) at the desired final concentration.

For SERS experiments, a colloidal solution of cetrimonium bromide-capped gold nanobipyramids (AuBPs, 1  mg/ml in water) having a diameter and length of 35 and 105 nm, respectively, was used (Nanoseedz, Hong Kong). Milli-Q water was used throughout our experiments. Fresh solutions of biomolecules were considered for the measurements.

### 2.2 Optical Fiber-Based Light Delivery System for Raman Measurements

**Fig. 1:**
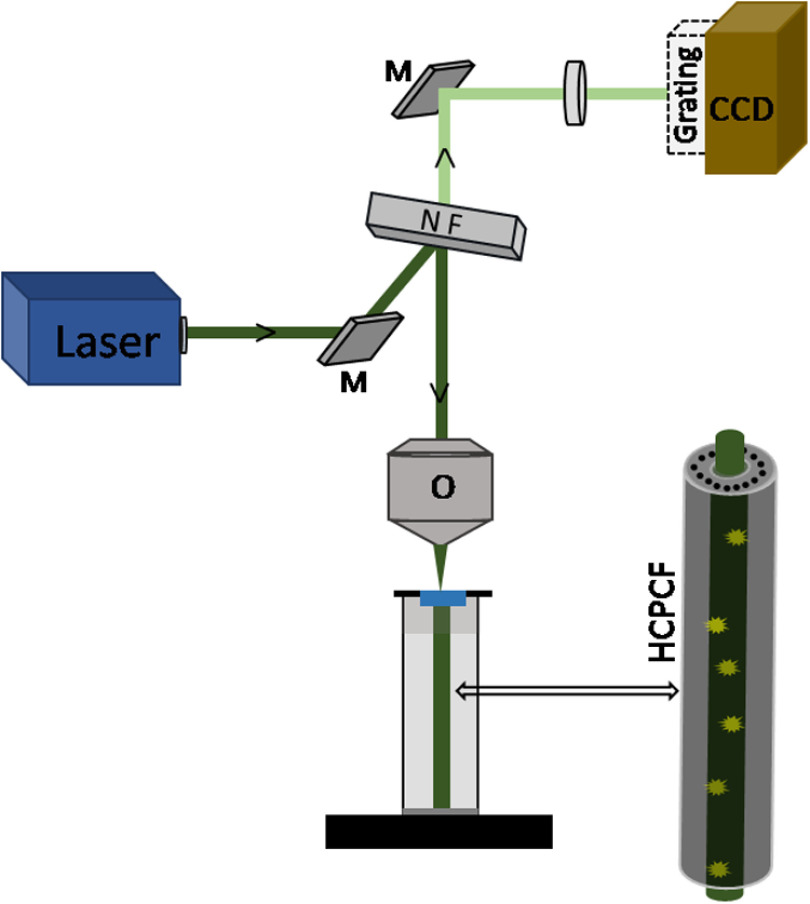
Schematic of the fiber-enhanced Raman spectroscopic setup. (M, mirror; NF, notch filter; and O, microscopic objective).

An HCPCF-based light delivery system was assembled for carrying out the Raman measurements. The optical setup consisted of a commercial confocal Raman microspectrometer (Labram HR, Horiba Jobin-Yvon, Japan) coupled with a homemade fiber holder that holds the HCPCF (Fig. 1). The excitation laser produced by a solid-state diode laser source emitting at 532 nm was focused into the fiber core using a 50× long working distance microscope objective (NA 0.55, Olympus). The Raman scattered light from the sample propagated back through the fiber and was collected in a backscattering geometry using the same objective, was analyzed by a diffraction grating (600  grooves/mm), and acquired by a charged coupled device (Synapse CCD Detector) embedded in the spectrometer.

All experiments were performed with an incident power measured on the sample of 5 mW, whereas the spectral acquisition times for ethanol, Mb, and Aβ42 were 5, 10, and 15 s, respectively. Before measurements, the spectroscopic system was calibrated on the $520.8\;{\mathrm{cm}}^{-1}$ Raman peak of a silicon wafer.

Commercial hollow core crystal fibers (Model HC-1060, Thorlabs) were used in FERS experiments. Both ends of a 5-cm-long fiber were first cleaved, then the fiber was meticulously vertically aligned to the microscope objective using a fiber holder mounted on an XYZ stage. The chamber of the fiber holder was filled with the sample solution (30  μl), which was then allowed to flow down through the microcavities of the fiber for 2 min before the measurements. Photodegradation of the analytes during Raman spectra acquisition was cautiously prevented by assuring a smooth continuous flow of analyte solution through the fiber during the measurements. This was achieved by pumping the analyte solution using a syringe pump in to the sample holder at a regular interval of time. Control measurements were carried out by direct Raman sampling, in which the HCPCF was replaced with an open cuvette filled with the analyte solution and the laser was focused onto the exposed solution in the open cuvette.

## 3 Results and Discussion

### 3.1 Fiber-Enhanced Raman Sensing

**Fig. 2:**
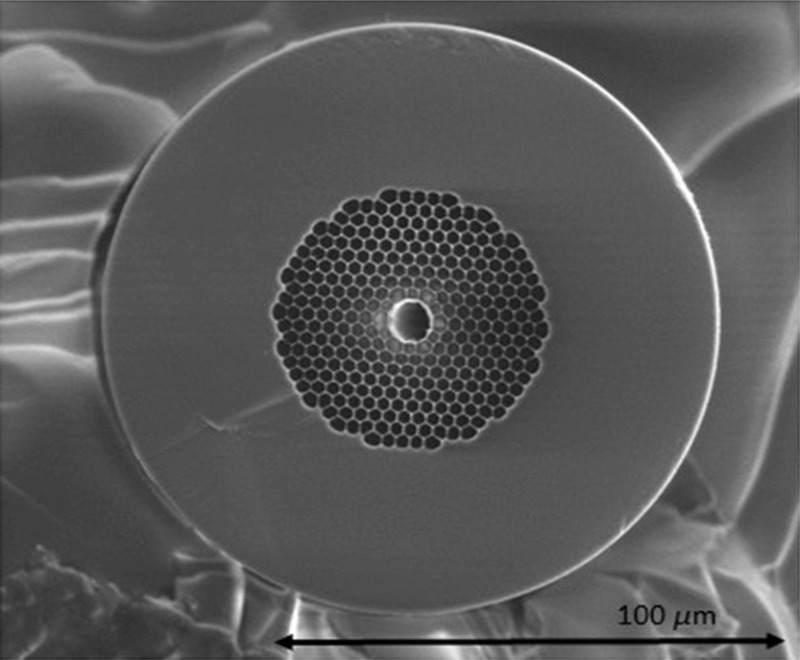
SEM image of the cleaved end of the HCPCF used in the experiments.

In order to compare the vibrational signal response produced by our FERS system with that obtained by a conventional Raman spectroscopy setup, Raman measurements were carried out both with the analyte solution inside the HCPCF and with a conventional direct sampling method. The HCPCF (Fig. 2) used in the experiments shows a core diameter of 10  μm, and its pitch diameter is about 2.6  μm. The microstructured air holey cladding region is periodically arranged around the central core with a total dimension of about 50  μm with an air filling fraction of >90%.

**Fig. 3:**
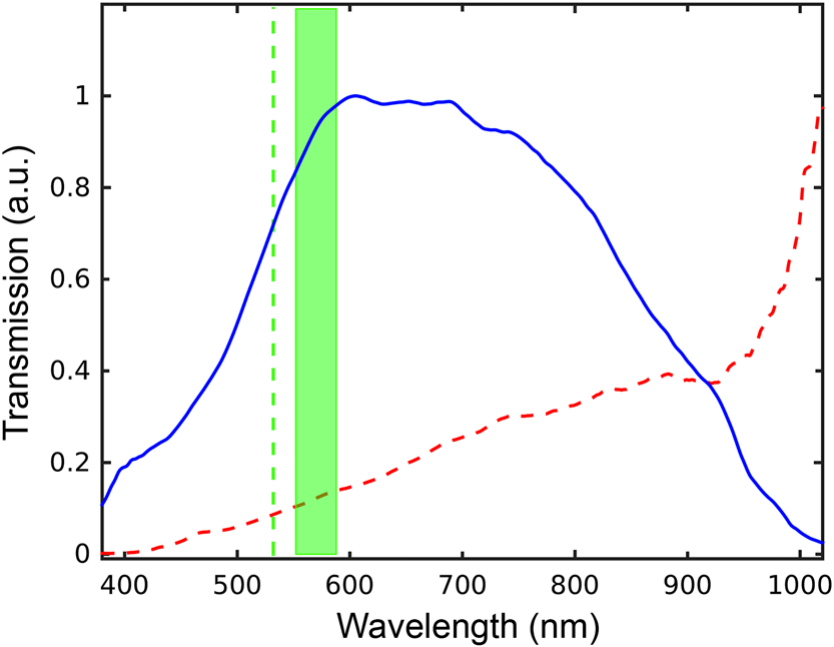
Transmission spectra of the HCPCF filled with air (red dashed line) and with water (blue continuous line). The excitation line at 532 nm (green dashed line) and the spectral range (green band) considered in our Raman experiments are also shown.

The background Raman spectrum of the optical fiber was preliminarily measured in order to assess the absence of any interference with the Raman spectra of the analytes. We verified that once the HCPCF is filled with water, due to a change in the refractive index, its transmission spectrum gets shifted from NIR (centerd at 1060 nm) to the visible range with maximum transmission in the region from 500 to 900 nm^36^ (Fig. 3).

### 3.2 FERS of Ethanol and Myoglobin

**Fig. 4:**
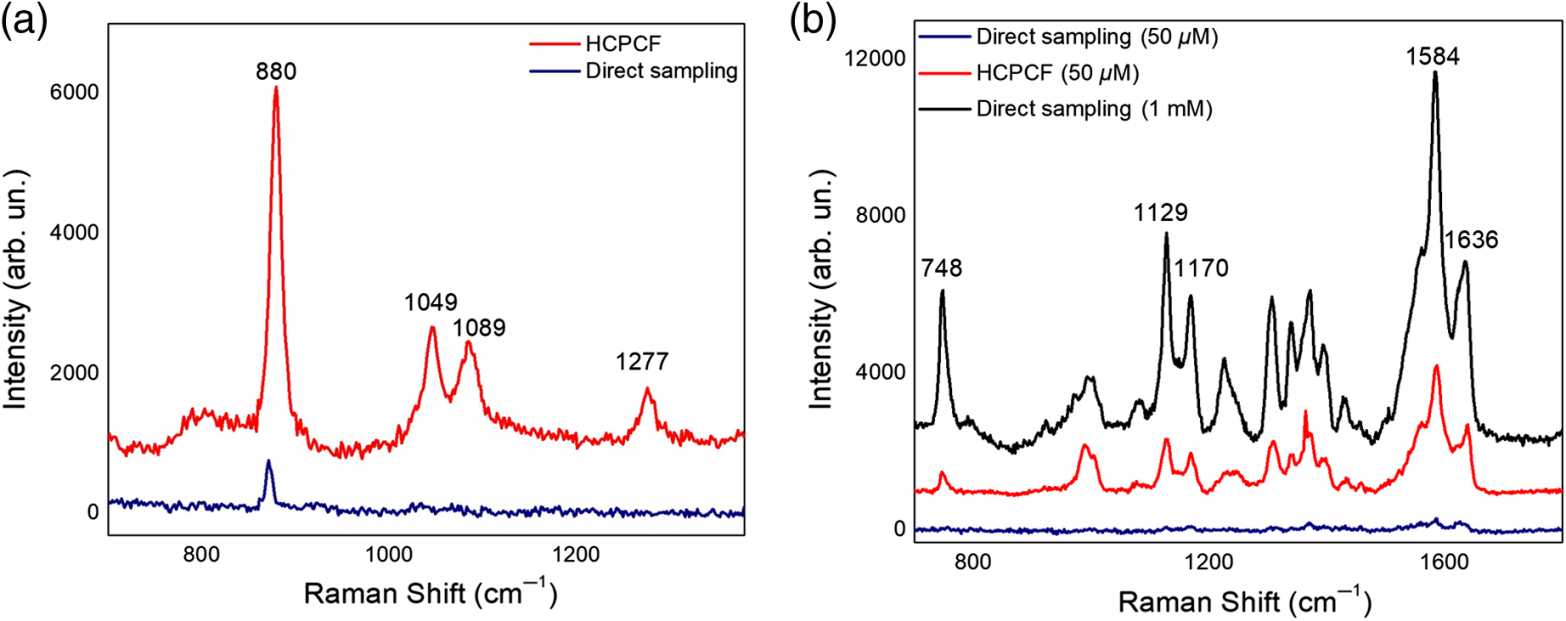
Comparison between Raman spectra acquired by direct sampling (blue, black lines) and using the HCPCF setup (red lines) of (a) a 25% ethanol solution and (b) Mb at 1 mM (black lines) and 50  μM (blue, red lines).

Initially, the Raman spectrum of a 25% (v/v) ethanol–water solution was recorded. Figure 4(a) shows a comparison between the Raman spectral profiles of ethanol as obtained by direct and HCPCF-mediated sampling. The Raman peak at $880\;{\mathrm{cm}}^{-1}$, which is attributed to the skeletal CCO symmetric stretching^36^^,^^47^ of ethanol, appears in both spectra. Nevertheless, compared to the sample measured inside the HCPCF, its peak intensity collected by conventional sampling appeared 10-fold lower. Moreover, the Raman peaks at 1049, 1089, and $1277\;{\mathrm{cm}}^{-1}$, which are assigned to skeletal CCO deformations, CO stretching, and deformation wagging of ethanol, respectively, were only detected in the sample measured in HCPCF. Subsequently, in order to test the system performances for biomolecule analysis, we examined the vibrational spectra of Mb, here taken as a model of a biomolecule extensively investigated in Raman studies^48^^,^^49^ [Fig. 4(b)]. Prominent vibrational peaks at 1129, 1170, 1306, 1372, 1584, and $1636\;{\mathrm{cm}}^{-1}$, which are attributed to υ22, υ30, υ21, υ4, υ19, and υ10 of the heme group, respectively,^48^^,^^50^ were observed by direct sampling of a highly concentrated (1 mM) protein solution. Further measurements were carried out by decreasing the protein concentration down to 50  μM. Similar to the case of ethanol, intense Raman signals were collected by FERS, while most of them were undistinguishable or noisy in the spectrum obtained by the conventional Raman scheme. These results offered us a first test bench proving the efficiency of our FERS-based setup.

### 3.3 FERS of Aβ42

**Fig. 5:**
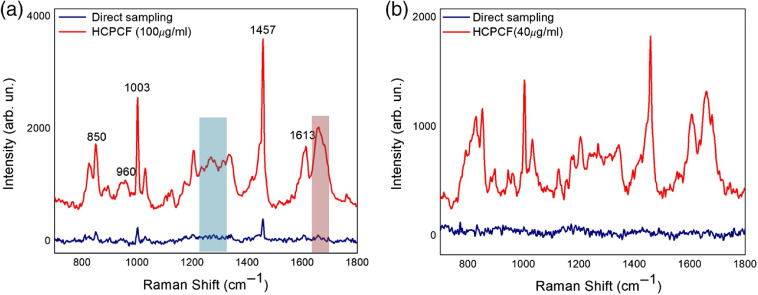
Raman spectra of Aβ42 acquired by conventional Raman spectroscopy (blue lines) and with an HCPCF (red lines): (a) 100  μg/ml and (b) 40  μg/ml. Shaded regions identify amide I (red) and amide III (blue) bands of Aβ.

Once we clarified the potential offered by FERS with HCPCF, we verified its efficacy in the Raman detection of Aβ42. Initially, measurements of Aβ with a concentration of 100  μg/ml were performed both under direct sampling and by the HCPCF-based setup [Fig. 5(a)]. Characteristic Raman modes of Aβ peaking at 830/850, 1003, 1457, and $1660\;{\mathrm{cm}}^{-1}$ can be identified by both approaches and ascribed to the Fermi doublet of Tyr, the aromatic Phe ring breathing, ${\mathrm{CH}}_{2}$ scissoring vibrations, and the amide I region, respectively^44^^–^^48^ (Table 1). Additional peaks were detected in the FERS spectrum ascribed to the backbone CCN stretching vibration ($960\;{\mathrm{cm}}^{-1}$), the amide III band (1230 to $1300\;{\mathrm{cm}}^{-1}$), and the aromatic Tyr ring stretching ($1613\;{\mathrm{cm}}^{-1}$). In accordance with previous results on ethanol and Mb, the spectral features of Aβ measured using HCPCF appeared sharp and intense in such a way that a signal enhancement of ∼20-fold at the minimum was observed as compared to the direct sampling modality. Nonetheless, apart from some exceptions (e.g., amide I, amide III, the ${850\text{-}\mathrm{cm}}^{-1}$ band of Tyr, and the ${1003\text{-}\mathrm{cm}}^{-1}$ band of Phe), the peaks of the Aβ solution measured by conventional Raman showed a low signal-to-noise ratio or were mostly imperceptible. These outcomes validated the potential of FERS with HCPCF for efficient detection of Aβ.

**Table 1:** Assignments of main Raman peaks of Aβ42.^49^

Peak position (${\mathrm{cm}}^{-1}$)	Mode assignment
830	Tyr
850	Tyr
960	CC str
1003	Phe
1030	Phe
1130	CN str
1205 to 1210	Phe/Tyr
1230 to 1270	Amide III
1455 to 1465	CH def
1530	His/amide II
1613	Tyr
1660 to 1690	Amide I

Afterward, we spent effort in demonstrating the sensitivity of our FERS setup upon decreasing the Aβ concentration. Successive FERS measurements were performed on a 40-μg/ml solution of Aβ [Fig. 5(b)]. In this case, the vibrational spectra measured with the FERS system appeared strongly enhanced compared to those obtained by conventional Raman, which were noisy without any distinct Raman peaks detected.

**Fig. 6:**
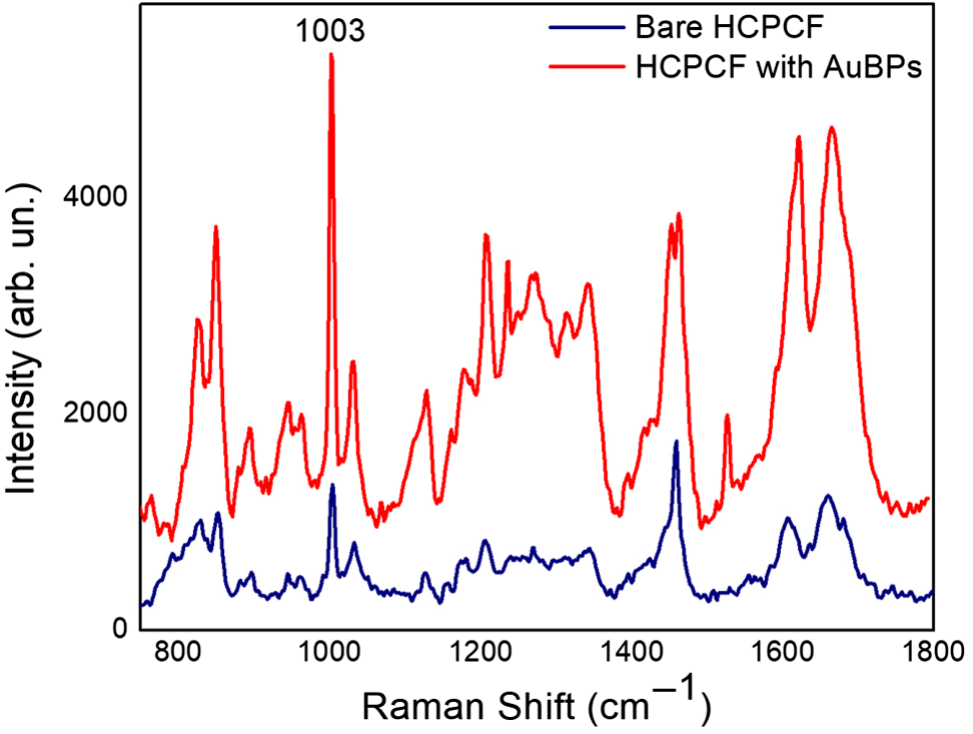
Raman spectra of 40  μg/ml Aβ42 achieved with a bare HCPCF (blue line) and with the AuBPs-coated HCPCF (red line).

Aimed at further improving the optical performances of our setup, we integrated the FERS system with AuBPs within the fiber core to take advantage of a supplementary SERS effect. The choice of using nonspherical nanoparticles here is motivated because of their well-established superior SERS efficiency generated by effective SERS hot spots at the sharp ends.^51^^,^^52^ AuBPs were adhered to the fiber core by electrostatic-mediated surface immobilization between positively charged nanoparticles and the negative surface charge of the inner silica core of the fiber. In short, the nanoparticle solution was allowed to enter into the fiber core by dipping the HCPCF in 2 ml of particle solution for about 5 min. The fiber was then dried at 40°C in an oven for 5 min to remove the solvent and extensively rinsed with Milli-Q water to remove unbound nanoparticles. The Aβ solution (40  μg/ml) was then allowed to flow through the particle-coated fiber core as in the previous measurements. Figure 6 depicts a comparison between spectra obtained by SERS-active HCPCF and bare HCPCF. We demonstrated that an additional ∼10 times signal enhancement could be attained using the AuBPs-functionalized fiber. All the prominent characteristic Raman peaks of Aβ were identified in the SERS spectrum, suggesting that the native structure of the inspected species was almost preserved.^53^ The appearance of prevailing peaks as, e.g., Tyr modes at 830, 850, and $1613\;{\mathrm{cm}}^{-1}$, Phe modes at 1003 and $1205\;{\mathrm{cm}}^{-1}$, as well as C─N vibrational modes from aminoterminated amino acids within the 1080-to ${1150\text{-}\mathrm{cm}}^{-1}$ region may represent an indication of the spatial proximity of these residues to the Au surface.^49^ An overall signal amplification of ∼200 times can be finally estimated as provided by both the use of a HCPCF and its integration with AuBPs within our Raman setup.

This work represents a proof-of-concept demonstration of an effective Raman detection system of Aβ42 showing high sensitivity combined with well-resolved and intense spectral Aβ42 features. Thanks to the above characteristics, the proposed FERS-SERS method proves suitable for future implementations aimed at extending the limit of detection to match physiological requirements. Aβ42 in the CSF of healthy humans ranges around 1.2±0.4  ng/ml and decreases in the case of AD patients.^54^^,^^55^ Future efforts will be spent to optimize the system by a comparison among different plasmonic nanoparticles, a tight control over their density, as well as their possible functionalization with biorecognition elements.

## 4 Conclusion

We demonstrated the advantages in supplying a standard Raman setup with a fiber-based Raman spectroscopic system for the analysis of molecules of biological and biomedical interest, including Aβ peptide as an established biomarker of AD. The measurements were performed using a HCPCF with a laser excitation wavelength of 532 nm. Increased interaction between the analyte and the guided laser excitation light as obtained inside an HCPCF leads to a flexible, reliable, and sensitive tool for the enhancement of weak Raman signals, such as those usually encountered with biomolecules. The results attested that, compared to the conventional direct sampling method, the FERS approach can generate a ≥20 times amplified signal from protein species. Furthermore, by equipping the HCPCF with plasmonic nanoparticles, the Raman signal can undergo an overall ∼200 times enhancement as a result of a supplementary SERS effect. Finally, advantages such as low sampling volume, the possibility of *in situ* measurements, flexibility, portability, and cost-effectiveness depict the proposed platform as particularly attractive in view of a preclinical or clinical detection of Aβ, paving the way toward an early detection of Alzheimer’s. Further studies will be aimed at verifying the potential of different metal nanoparticles and their tailored assembly inside the fiber core in further implementing the platform when using reduced concentration values of the analyte.
